# Recent advances in understanding anorexia nervosa

**DOI:** 10.12688/f1000research.17789.1

**Published:** 2019-04-17

**Authors:** Guido K.W. Frank, Megan E. Shott, Marisa C. DeGuzman

**Affiliations:** 1 Department of Psychiatry, University of Colorado, Anschutz Medical Campus, Aurora, CO, 80045, USA; 2 Neuroscience Program, University of Colorado, Anschutz Medical Campus, Aurora, CO, 80045, USA

**Keywords:** Anorexia nervosa, brain imaging, reward, learning, habit, brain, structure, function, behavior

## Abstract

Anorexia nervosa is a complex psychiatric illness associated with food restriction and high mortality. Recent brain research in adolescents and adults with anorexia nervosa has used larger sample sizes compared with earlier studies and tasks that test specific brain circuits. Those studies have produced more robust results and advanced our knowledge of underlying biological mechanisms that may contribute to the development and maintenance of anorexia nervosa. It is now recognized that malnutrition and dehydration lead to dynamic changes in brain structure across the brain, which normalize with weight restoration. Some structural alterations could be trait factors but require replication. Functional brain imaging and behavioral studies have implicated learning-related brain circuits that may contribute to food restriction in anorexia nervosa. Most notably, those circuits involve striatal, insular, and frontal cortical regions that drive learning from reward and punishment, as well as habit learning. Disturbances in those circuits may lead to a vicious cycle that hampers recovery. Other studies have started to explore the neurobiology of interoception or social interaction and whether the connectivity between brain regions is altered in anorexia nervosa. All together, these studies build upon earlier research that indicated neurotransmitter abnormalities in anorexia nervosa and help us develop models of a distinct neurobiology that underlies anorexia nervosa.

Anorexia nervosa (AN) is characterized by a persistent restriction of energy intake and leads to a body weight that is significantly lower than what is expected for height and age ^1^. There is either an intense fear of gaining weight or becoming fat or persistent behavior that interferes with weight gain (even though at significantly low weight). Individuals with AN experience a disturbance in the way one’s body weight or shape is experienced, undue influence of body shape and weight on self-evaluation, or persistent lack of recognition of the seriousness of the current low body weight. A restricting type has been distinguished from a binge eating/purging type; individuals in the latter group may intermittently have binge eating episodes or may use self-induced vomiting to avoid weight gain. AN shows a complex interplay between neurobiological, psychological, and environmental factors ^2^ and is a chronic disorder with frequent relapse, high treatment costs, and severe disease burden ^3, 4^. AN has a mortality rate 12 times higher than the death rate for all causes of death for females 15 to 24 years old ^5– 7^. Treatment success is modest, and no medication has been approved for AN treatment ^8^.

Various psychological or psychodynamic theories have been developed in the past to explain the causes of AN but their underlying theories have been difficult to test ^9^. On the contrary, neurobiological research using techniques such as human brain imaging leads to more directly testable hypotheses and holds promise to help us tease apart mechanisms that contribute to the onset of the illness, maintenance of AN behavior, and recovery from AN. This article will review recent advances in our understanding of the neurobiology of AN. Neurobiology is a branch of the life sciences, which deals with the anatomy, physiology, and pathology of the nervous system ^10^. Neurobiology is closely associated with the field of neuroscience, a branch of biology, which tries to understand brain function, from gross anatomy to neural circuits and cells that comprise them ^11^. The goal of neurobiological research in AN is to develop a medical model perspective to reduce stigma and help develop better treatments ^12^. At the earlier stages of brain research in AN, study samples tended to be quite small, which made replication difficult ^13^. Most frequently, altered serotonin function was associated with AN and anxiety in the disorder ^14^. More recent brain research has built upon those studies and increased sample sizes in structural studies and introduced studying brain function in relation to specific tasks that are thought be related to food restriction, anxiety, and body image distortion. Most studies have been carried out in adults, although there is a growing body of literature that investigated youth with AN.

The most frequently applied brain imaging study design in the past studied brain volume in AN, and more recent research now allows cortical thickness of the brain to be investigated. For a long time, there was the notion that gray matter volume and cortical thickness are lower in patients with AN (when ill and after recovery) than in controls. This research was pioneered by Katzman *et al*. in adolescents with AN ^15, 16^. However, recent research by Bernardoni *et al*. ^17^ and King *et al*. ^18^ in adolescents and young adults indicated that such abnormalities are rather short-lived and that both lower volume and cortical thickness normalize with weight recovery. Animal studies suggest that those changes may be due to the effects of malnutrition and dehydration on astrocytes within the brain connective tissue ^19^. Two studies from our group have found larger orbitofrontal cortex and insula volume in adults and adolescents with AN after 1 to 2 weeks of normalization of food intake or in individuals after recovery, and orbitofrontal cortex volume was related to taste pleasantness ^20, 21^. Those results were intriguing as they implicated taste perception in relation to brain volume but they need replication. New data from our group in healthy first-degree relatives of patients with AN also show larger orbitofrontal cortex volume, supporting a trait abnormality (unpublished data). Studies by Bernardoni *et al*. in young adults have found abnormalities in gray matter gyrification in AN, and nutritional rehabilitation seems to normalize altered cortical folding ^22^. A valuable lesson from those studies is that food intake can have dramatic effects on brain structure. Whether lower or higher brain volume in AN has implications on illness behavior or is instead an effect of malnutrition without effects on behavior is still unclear and needs further research ^23, 24^.

Functional brain imaging provides the opportunity to tie behavior to brain activation and thus to distinct brain neurobiology, which could become a treatment target. Several aspects of behavior in AN stand out. One is the ability to restrict food intake to the point of emaciation while the typical mechanisms to maintain a healthy body weight are inefficient. Another is how the body can maintain this behavior even when AN patients in therapy are trying to break that behavior pattern.

Relevant to food avoidance behavior is the brain reward system, which processes the motivation to eat and hedonic experience after food intake, and also calculates and updates how valuable a specific food is to us ^25^. This circuitry includes the insula, which contains the primary taste cortex, the ventral striatum that comprises dopamine terminals to drive food approach, and the orbitofrontal cortex that calculates a value, while the hypothalamus integrates body signals on hunger and satiety for higher-order decision making and food approach. Many studies have used *visual* food cues but it has been difficult to draw conclusions on the pathophysiology of AN from those studies ^26^.

Several studies from our group using sugar taste stimuli have found that brain activation in adolescent and adult AN was elevated compared with controls in response to unexpected receipt or omission of sweet taste in the insula and striatum ^27, 28^. This so-called “prediction error” response has been associated with brain dopamine circuitry and serves as a learning signal to drive approach or avoidance of salient stimuli in the environment in the future. In addition, orbitofrontal cortex prediction error response correlated positively with anxiety measures in AN ^28, 29^. We found a similar pattern of elevated brain activation in AN to unexpected receipt or omission of monetary stimuli, suggesting a food-independent alteration of brain dopamine circuitry. Importantly, those studies have also shown that brain response was predictive of weight gain during treatment and that brain dopamine function could have an important role in weight recovery in AN. This was supported by a retrospective chart review in adolescents with AN that suggested that the dopamine D _2_ receptor partial agonist aripiprazole was associated with higher weight gain in a structured treatment program in comparison with patients not on that medication ^30^. Mechanistically, it was hypothesized that dopamine D _2_ receptor stimulation might be desensitizing those receptors and normalize behavior response. This, however, is speculative and controlled studies are lacking.

Other lines of research on the pathophysiology of AN are directed toward feedback learning, and several studies have found that AN is associated with alterations, behaviorally or in brain response. A study by Foerde and Steinglass, who investigated learning using a picture association task in patients with AN before and after weight restoration, indicated deficits in feedback learning and generalization of learned information in comparison with controls ^31^. Such alterations could translate directly into difficulties in behavior modification toward recovery. Studies from Ehrlich’s group found normal feedback learning in ill, but reduced performance on reversal learning in recovered AN, which made the impact of learning in ill AN less clear ^32, 33^. Furthermore, Bernardoni *et al*., using a different study design, found that individuals with AN had an *increased* learning rate and elevated medial frontal cortex response following punishment ^34^. That result supports previous findings of elevated sensitivity to punishment in AN as a possible biological trait ^35^. Another very interesting study by Foerde *et al*. tested brain response to food choice presenting images of food and that research implicated the dorsal striatum in this process in AN ^36^. The authors also found that the strength of connectivity between striatum and frontal cortex activation correlated inversely with actual caloric food intake in a test meal after the brain scan. The authors interpreted the findings to mean that this frontostriatal involvement in AN could contribute to habit formation of food restriction behavior. Behavioral research has provided evidence that habit formation or habit strength could be necessary for the perpetuation of AN behaviors and this concept is important to study further ^37– 39^.

The self-perception of being fat despite being underweight is another aspect of AN that the field continues to struggle with in finding its underlying pathophysiology. Some studies have found a specific brain response related to altered processing of visual information or tasks that tested interoception. For instance, Kerr *et al*. ^40^ found elevated insula activation during an abdomen perception task, and Xu *et al*. ^41^ found that a frontal and cingulate cortex response during a social evaluation task correlated with body shape concerns. A study by Hagman *et al*., however, indicated a strong cognitive and emotional influence on body image distortion, and the intersection between altered perception and fear-driven self-perception needs further study ^42^. Social interaction and its brain biology constitute another area that was hypothesized to be related to AN behaviors and some research is emerging on this topic. For instance, a study by McAdams *et al*. showed that the quality of the social relationship or social reciprocity tested in a trust game showed lower occipito-parietal brain response in patients with AN in comparison with a control group ^43^. This research suggests altered reward experience from interpersonal contact in AN, which could impact emotional well-being and interfere with recovery. Oxytocin, a peptide hormone related to social behavior, could play a role but this requires more detailed research ^44^.

Studies on brain connectivity can test either what brain regions work in concert during a specific task (functional connectivity) or what the hierarchical organization is between areas in the brain (that is, what region drives another) (effective connectivity). Several studies in the past have shown that resting-state functional connectivity is altered in patients with AN compared with control groups. Those studies repeatedly found altered connectivity that involved the insula, a region associated with taste perception, prediction error processing, and integration of body perception, as reviewed by Gaudio *et al*. ^45^. More recent studies found higher or lower resting-state activation in AN across various networks and during rest or task conditions ^39, 46– 49^. Longitudinal studies will need to test what might be the best resting-state network to focus on to predict, for instance, illness outcome or whether functional connectivity during specific tasks such as taste processing could be more informative. One study by Boehm *et al*. found normalization of functional connectivity in the default mode but continued abnormal frontoparietal network connectivity in recovered AN ^50^. It remains to be seen whether functional connectivity will normalize with recovery or can identify long-lasting or maybe trait alterations.

Effective connectivity studies indicated that while viewing fearful faces, a group with AN had deficits of brain connectivity between prefrontal cortex and the amygdala, which correlated with measures for anxiety and eating behaviors in a study by Rangaprakash *et al*. ^51^. Studies from our group that assessed effective connectivity during the tasting of sucrose solution found that, whereas in controls the hypothalamus drove ventral striatum response, in patients with AN, effective connectivity was directed from the ventral striatum to the hypothalamus ^28, 52^. Previously, a dopamine-dependent pathway from the ventral striatum to the hypothalamus that mediates fear was described and we hypothesized that this circuitry might be activated in AN to override appetitive hypothalamic signals ^53^.

In summary, brain research has started to make inroads into the pathophysiology of AN. We have learned that malnutrition has significant effects on brain structure, changes that can recover with weight restoration, but whether those alterations have an impact on illness behavior remains unclear ^23^. Research into the function of brain circuits has implicated reward pathways and malnutrition-driven alterations of dopamine responsiveness together with neuroendocrine changes, and high anxiety may interfere with normal mechanisms that drive eating behavior ^54^. Habit learning and associated striatal-frontal brain connectivity could provide another mechanism of how brain function and interaction of cortical and sub-cortical regions may perpetuate illness behavior that is difficult to overcome. Those advances are promising to establish that AN is associated with a distinct brain pathophysiology. This will help researchers develop effective biological treatments that improve recovery and help prevent relapse. A significant challenge to overcome will be to integrate the differing brain research studies and develop a unified model ^13^. Critical in this effort will be well-powered and comparable study designs across research groups, which take into account confounding factors such as comorbidity and medication use and which use rigorous standards for data analysis.

## References

[1] American Psychiatric Association: Desk reference to the diagnostic criteria from DSM-5. Washington, DC: American Psychiatric Publishing.2013 Reference Source

[2] Bulik CM: Exploring the gene-environment nexus in eating disorders. *J Psychiatry Neurosci.* 2005;30(5):335–9. 16151538 PMC1197278

[3] Khalsa SS Portnoff LC McCurdy-McKinnon D: What happens after treatment? A systematic review of relapse, remission, and recovery in anorexia nervosa. *J Eat Disord.* 2017;5:20. 10.1186/s40337-017-0145-3 28630708 PMC5470198

[4] Hay P Mitchison D Collado AEL: Burden and health-related quality of life of eating disorders, including Avoidant/Restrictive Food Intake Disorder (ARFID), in the Australian population. *J Eat Disord.* 2017;5:21. 10.1186/s40337-017-0149-z 28680630 PMC5494787

[5] Golden NH: Eating disorders in adolescence and their sequelae. *Best Pract Res Clin Obstet Gynaecol.* 2003;17(1):57–73. 10.1053/ybeog.2003.0344 12758226

[6] Rosling AM Sparén P Norring C: Mortality of eating disorders: a follow-up study of treatment in a specialist unit 1974-2000. *Int J Eat Disord.* 2011;44(4):304–10. 10.1002/eat.20827 21472749

[7] Sullivan PF: Mortality in anorexia nervosa. *Am J Psychiatry.* 1995;152(7):1073–4. 10.1176/ajp.152.7.1073 7793446

[8] Frank GK Shott ME: The Role of Psychotropic Medications in the Management of Anorexia Nervosa: Rationale, Evidence and Future Prospects. *CNS Drugs.* 2016;30(5):419–42. 10.1007/s40263-016-0335-6 27106297 PMC4873415

[9] Lutter M Croghan AE Cui H: Escaping the Golden Cage: Animal Models of Eating Disorders in the Post-Diagnostic and Statistical Manual Era. *Biol Psychiatry.* 2016;79(1):17–24. 10.1016/j.biopsych.2015.02.006 25777657

[10] Merriam-Webster Inc: The Merriam-Webster dictionary. New edition. ed. Springfield, Massachusetts: Merriam-Webster, Incorporated.2016 Reference Source

[11] Kandel ER: Principles of neural science. 5th ed. New York: McGraw-Hill.2013 Reference Source

[12] Vengeliene V Bespalov A Roßmanith M: Towards trans-diagnostic mechanisms in psychiatry: neurobehavioral profile of rats with a loss-of-function point mutation in the dopamine transporter gene. *Dis Model Mech.* 2017;10(4):451–61. 10.1242/dmm.027623 28167616 PMC5399565

[13] Frank GKW Favaro A Marsh R: Toward valid and reliable brain imaging results in eating disorders. *Int J Eat Disord.* 2018;51(3):250–61. 10.1002/eat.22829 29405338 PMC7449370

[14] Kaye WH Wierenga CE Bailer UF: Nothing tastes as good as skinny feels: the neurobiology of anorexia nervosa. *Trends Neurosci.* 2013;36(2):110–20. 10.1016/j.tins.2013.01.003 23333342 PMC3880159

[15] Katzman DK Lambe EK Mikulis DJ: Cerebral gray matter and white matter volume deficits in adolescent girls with anorexia nervosa. *J Pediatr.* 1996;129(6):794–803. 10.1016/S0022-3476(96)70021-5 8969719

[16] Katzman DK Zipursky RB Lambe EK: A longitudinal magnetic resonance imaging study of brain changes in adolescents with anorexia nervosa. *Arch Pediatr Adolesc Med.* 1997;151(8):793–7. 10.1001/archpedi.1997.02170450043006 9265880

[17] Bernardoni F King JA Geisler D: Weight restoration therapy rapidly reverses cortical thinning in anorexia nervosa: A longitudinal study. *Neuroimage.* 2016;130:214–22. 10.1016/j.neuroimage.2016.02.003 26876474

[18] King JA Geisler D Ritschel F: Global cortical thinning in acute anorexia nervosa normalizes following long-term weight restoration. *Biol Psychiatry.* 2015;77(7):624–32. 10.1016/j.biopsych.2014.09.005 25433902

[19] Frintrop L Liesbrock J Paulukat L: Reduced astrocyte density underlying brain volume reduction in activity-based anorexia rats. *World J Biol Psychiatry.* 2018;19(3):225–35. 10.1080/15622975.2016.1273552 28132573

[20] Frank GK Shott ME Hagman JO: Alterations in brain structures related to taste reward circuitry in ill and recovered anorexia nervosa and in bulimia nervosa. *Am J Psychiatry.* 2013;170(10):1152–60. 10.1176/appi.ajp.2013.12101294 23680873 PMC3789862

[21] Frank GK Shott ME Hagman JO: Localized brain volume and white matter integrity alterations in adolescent anorexia nervosa. *J Am Acad Child Adolesc Psychiatry.* 2013;52(10):1066–1075.e5. 10.1016/j.jaac.2013.07.007 24074473 PMC4082770

[22] Bernardoni F King JA Geisler D: Nutritional Status Affects Cortical Folding: Lessons Learned From Anorexia Nervosa. *Biol Psychiatry.* 2018;84(9):692–701. 10.1016/j.biopsych.2018.05.008 29910027

[23] Frank GK: What causes eating disorders, and what do they cause? *Biol Psychiatry.* 2015;77(7):602–3. 10.1016/j.biopsych.2015.01.012 25766685

[24] King JA Frank GKW Thompson PM: Structural Neuroimaging of Anorexia Nervosa: Future Directions in the Quest for Mechanisms Underlying Dynamic Alterations. *Biol Psychiatry.* 2018;83(3):224–34. 10.1016/j.biopsych.2017.08.011 28967386 PMC6053269

[25] Kelley AE: Ventral striatal control of appetitive motivation: role in ingestive behavior and reward-related learning. *Neurosci Biobehav Rev.* 2004;27(8):765–76. 10.1016/j.neubiorev.2003.11.015 15019426

[26] Lloyd EC Steinglass JE: What can food-image tasks teach us about anorexia nervosa? A systematic review. *J Eat Disord.* 2018;6:31. 10.1186/s40337-018-0217-z 30410758 PMC6211517

[27] Frank GK Reynolds JR Shott ME: Anorexia nervosa and obesity are associated with opposite brain reward response. *Neuropsychopharmacology.* 2012;37(9):2031–46. 10.1038/npp.2012.51 22549118 PMC3398719

[28] Frank GKW DeGuzman MC Shott ME: Association of Brain Reward Learning Response With Harm Avoidance, Weight Gain, and Hypothalamic Effective Connectivity in Adolescent Anorexia Nervosa. *JAMA Psychiatry.* 2018;75(10):1071–1080. 10.1001/jamapsychiatry.2018.2151 30027213 PMC6233809

[29] Schultz W: Getting formal with dopamine and reward. *Neuron.* 2002;36(2):241–63. 10.1016/S0896-6273(02)00967-4 12383780

[30] Frank GK Shott ME Hagman JO: The partial dopamine D2 receptor agonist aripiprazole is associated with weight gain in adolescent anorexia nervosa. *Int J Eat Disord.* 2017;50(4):447–50. 10.1002/eat.22704 28334444 PMC5392387

[31] Foerde K Steinglass JE: Decreased feedback learning in anorexia nervosa persists after weight restoration. *Int J Eat Disord.* 2017;50(4):415–23. 10.1002/eat.22709 28393399 PMC5869029

[32] Geisler D Ritschel F King JA: Increased anterior cingulate cortex response precedes behavioural adaptation in anorexia nervosa. *Sci Rep.* 2017;7:42066. 10.1038/srep42066 28198813 PMC5304157

[33] Ritschel F Geisler D King JA: Neural correlates of altered feedback learning in women recovered from anorexia nervosa. *Sci Rep.* 2017;7(1):5421. 10.1038/s41598-017-04761-y 28710363 PMC5511172

[34] Bernardoni F Geisler D King JA: Altered Medial Frontal Feedback Learning Signals in Anorexia Nervosa. *Biol Psychiatry.* 2018;83(3):235–43. 10.1016/j.biopsych.2017.07.024 29025688

[35] Jappe LM Frank GK Shott ME: Heightened sensitivity to reward and punishment in anorexia nervosa. *Int J Eat Disord.* 2011;44(4):317–24. 10.1002/eat.20815 21472750 PMC3072848

[36] Foerde K Steinglass JE Shohamy D: Neural mechanisms supporting maladaptive food choices in anorexia nervosa. *Nat Neurosci.* 2015;18(11):1571–3. 10.1038/nn.4136 26457555 PMC4624561

[37] Coniglio KA Becker KR Franko DL: Won't stop or can't stop? Food restriction as a habitual behavior among individuals with anorexia nervosa or atypical anorexia nervosa. *Eat Behav.* 2017;26:144–7. 10.1016/j.eatbeh.2017.03.005 28388511

[38] Steinglass JE Glasofer DR Walsh E: Targeting habits in anorexia nervosa: a proof-of-concept randomized trial. *Psychol Med.* 2018;48(15):2584–91. 10.1017/S003329171800020X 29455696

[39] Haynos AF Hall LMJ Lavender JM: Resting state functional connectivity of networks associated with reward and habit in anorexia nervosa. *Hum Brain Mapp.* 2019;40(2):652–62. 10.1002/hbm.24402 30251758 PMC6314844

[40] Kerr KL Moseman SE Avery JA: Altered Insula Activity during Visceral Interoception in Weight-Restored Patients with Anorexia Nervosa. *Neuropsychopharmacology.* 2016;41(2):521–8. 10.1038/npp.2015.174 26084229 PMC5130127

[41] Xu J Harper JA Van Enkevort EA: Neural activations are related to body-shape, anxiety, and outcomes in adolescent anorexia nervosa. *J Psychiatr Res.* 2017;87:1–7. 10.1016/j.jpsychires.2016.12.005 27978457 PMC5421407

[42] Hagman J Gardner RM Brown DL: Body size overestimation and its association with body mass index, body dissatisfaction, and drive for thinness in anorexia nervosa. *Eat Weight Disord.* 2015;20(4):449–55. 10.1007/s40519-015-0193-0 25929983

[43] McAdams CJ Lohrenz T Montague PR: Neural responses to kindness and malevolence differ in illness and recovery in women with anorexia nervosa. *Hum Brain Mapp.* 2015;36(12):5207–19. 10.1002/hbm.23005 26416161 PMC4715806

[44] Sala M Han K Acevedo S: Oxytocin Receptor Polymorphism Decreases Midline Neural Activations to Social Stimuli in Anorexia Nervosa. *Front Psychol.* 2018;9:2183. 10.3389/fpsyg.2018.02183 30542304 PMC6277875

[45] Gaudio S Wiemerslage L Brooks SJ: A systematic review of resting-state functional-MRI studies in anorexia nervosa: Evidence for functional connectivity impairment in cognitive control and visuospatial and body-signal integration. *Neurosci Biobehav Rev.* 2016;71:578–89. 10.1016/j.neubiorev.2016.09.032 27725172

[46] Olivo G Swenne I Zhukovsky C: Reduced resting-state connectivity in areas involved in processing of face-related social cues in female adolescents with atypical anorexia nervosa. *Transl Psychiatry.* 2018;8(1):275. 10.1038/s41398-018-0333-1 30546060 PMC6293319

[47] Spalatro AV Amianto F Huang Z: Neuronal variability of Resting State activity in Eating Disorders: Increase and decoupling in Ventral Attention Network and relation with clinical symptoms. *Eur Psychiatry.* 2019;55:10–7. 10.1016/j.eurpsy.2018.08.005 30384106

[48] Uniacke B Wang Y Biezonski D: Resting-state connectivity within and across neural circuits in anorexia nervosa. *Brain Behav.* 2019;9(1):e01205. 10.1002/brb3.1205 30590873 PMC6373651

[49] McFadden KL Tregellas JR Shott ME: Reduced salience and default mode network activity in women with anorexia nervosa. *J Psychiatry Neurosci.* 2014;39(3):178–88. 10.1503/jpn.130046 24280181 PMC3997603

[50] Boehm I Geisler D Tam F: Partially restored resting-state functional connectivity in women recovered from anorexia nervosa. *J Psychiatry Neurosci.* 2016;41(6):377–85. 10.1503/jpn.150259 27045551 PMC5082508

[51] Rangaprakash D Bohon C Lawrence KE: Aberrant Dynamic Connectivity for Fear Processing in Anorexia Nervosa and Body Dysmorphic Disorder. *Front Psychiatry.* 2018;9:273. 10.3389/fpsyt.2018.00273 29997532 PMC6028703

[52] Frank GK Shott ME Riederer J: Altered structural and effective connectivity in anorexia and bulimia nervosa in circuits that regulate energy and reward homeostasis. *Transl Psychiatry.* 2016;6(11):e932. 10.1038/tp.2016.199 27801897 PMC5314116

[53] Castro DC Cole SL Berridge KC: Lateral hypothalamus, nucleus accumbens, and ventral pallidum roles in eating and hunger: interactions between homeostatic and reward circuitry. *Front Syst Neurosci.* 2015;9:90. 10.3389/fnsys.2015.00090 26124708 PMC4466441

[54] Monteleone AM Castellini G Volpe U: Neuroendocrinology and brain imaging of reward in eating disorders: A possible key to the treatment of anorexia nervosa and bulimia nervosa. *Prog Neuropsychopharmacol Biol Psychiatry.* 2018;80(Pt B):132–42. 10.1016/j.pnpbp.2017.02.020 28259721

